# PREFACE

**DOI:** 10.2174/157015913804999450

**Published:** 2013-01

**Authors:** Tom Salt

This Editorial marks the beginning of the eleventh year of publication of ***Current Neuropharmacology***. The impact of *Current Neuropharmacology* is steadily increasing and this volume marks another important milestone as the journal increases to six issues per year. The standard and range of reviews and special issues consistently attains high standards. For Current Neuropharmacology to continue to thrive it is important that diversity and high standards are maintained. Of course, this depends on the dedicated efforts throughout the year of authors, Editorial Board members, Special Guest Editors, anonymous reviewers, and of course the efforts of Bentham Science Publishers. I express my thanks to all of you.

The diversity and high standard of work published in *Current Neuropharmacology* is reflected in the content of the present issue of the journal. A series of ‘Hot Topic’ papers on *Advances in Dystonia* is guest-edited by Christopher Bragg and Nutan Sharma. This is followed by a group of papers that reflects the diversity and wide scope of neuropharmacology. With its impact, visibility and availability on PubMed Central, the journal offers an excellent vehicle for the publication of novel reviews and hypotheses.

